# Effect of Water Vapor on Oxidation Processes of the Cu(111) Surface and Sublayer

**DOI:** 10.3390/ijms24010810

**Published:** 2023-01-03

**Authors:** Young Jae Kim, Daeho Kim, Yongman Kim, Yongchan Jeong, Beomgyun Jeong, Jeong Young Park

**Affiliations:** 1 Department of Chemistry, Korea Advanced Institute of Science and Technology (KAIST), Daejeon 34141, Republic of Korea; 2 Center for Nanomaterials and Chemical Reactions, Institute of Basic Science (IBS), Daejeon 34141, Republic of Korea; 3 Research Center for Materials Analysis, Korea Basic Science Institute (KBSI), Daejeon 34133, Republic of Korea

**Keywords:** oxidation reaction, copper, copper oxide, water, near-ambient techniques, operando observation, surface chemistry

## Abstract

Copper-based catalysts have different catalytic properties depending on the oxidation states of Cu. We report operando observations of the Cu(111) oxidation processes using near-ambient pressure scanning tunneling microscopy (NAP-STM) and near-ambient pressure X-ray photoelectron spectroscopy (NAP-XPS). The Cu(111) surface was chemically inactive to water vapor, but only physisorption of water molecules was observed by NAP-STM. Under O_2_ environments, dry oxidation started at the step edges and proceeded to the terraces as a Cu_2_O phase. Humid oxidation of the H_2_O/O_2_ gas mixture was also promoted at the step edges to the terraces. After the Cu_2_O covered the surface under humid conditions, hydroxides and adsorbed water layers formed. NAP-STM observations showed that Cu_2_O was generated at lower steps in dry oxidation with independent terrace oxidations, whereas Cu_2_O was generated at upper steps in humid oxidation. The difference in the oxidation mechanisms was caused by water molecules. When the surface was entirely oxidized, the diffusion of Cu and O atoms with a reconstruction of the Cu_2_O structures induced additional subsurface oxidation. NAP-XPS measurements showed that the Cu_2_O thickness in dry oxidation was greater than that in humid oxidation under all pressure conditions.

## 1. Introduction

Copper-based catalysts can exist in three well-known types of oxidation states: metallic Cu, Cu_2_O, and CuO. Each of the Cu oxidation states has different structural and electronic properties [1]. Controlling the oxidation state of Cu affects chemical reactivities and reaction mechanisms in catalytic reactions of copper-based materials [2]. Therefore, there have been many attempts to successfully oxidize and reduce the copper-based catalysts between Cu^0^, Cu^1+^, and Cu^2+^ [1,3,4]. Controlling not only the Cu oxidation state before the reactions, but also changes of Cu oxidation state during the reactions, is important to understand the catalytic properties of copper-based catalysts. The transitions of Cu oxidation states are common phenomena for many catalytic reactions of copper-based catalysts, such as water dissociation [5,6], low-temperature water–gas shift [7], CO oxidation [8,9], and CO_2_ hydrogenation [10,11]. For CO oxidation, the metallic Cu model catalyst is oxidized with a thin Cu_2_O layer, even under oxygen-lean conditions, which could affect the reaction mechanism [12].

The surface properties and reaction processes of Cu oxidation are crucial for investigating the relationship between the catalytic activity and the oxidation state of Cu-based catalysts. Although the surface energy of Cu(111) is lower than the other Cu facets, the Cu(111) surface has been used as a model catalyst to study the surface properties of an oxidized Cu surface [13]. In the initial stages of Cu(111) oxidation, step edges on the Cu(111) surface are a starting position for the oxidation process at room temperature (RT) [14]. When the Cu(111) is annealed in 10^−8^–10^−5^ Torr of O_2_ environments, “29” and “44” oxide layers, Cu_2_O-like structures, are formed on the surface at 423 K and 673 K, respectively [2,15,16]. The existence of 5–7 defects in the Cu_2_O surface on the Cu(111) was discovered as an intermediate structure before forming the “44” oxide layer [16]. The bulk Cu_2_O(111)/Cu(111) system and its intrinsic defects are also investigated by scanning tunneling microscopy (STM) [17]. Unveiling the complex structures of oxidized surface layers on the Cu(111) gives a fundamental understanding of the oxidation process on metallic copper materials. Operando direct observation under reaction conditions is required to elucidate the surface and properties of catalytic materials [18,19,20,21,22], such as the oxidized copper-based catalysts.

In this study, we investigated the operando oxidation processes of the Cu(111) under dry and humid O_2_ conditions, using near-ambient pressure measurements at RT. We first confirmed that the Cu(111) had no chemical interactions with water molecules by using near-ambient pressure STM (NAP-STM). In contrast, O_2_ molecules were dissociated at the step edges to oxidize the terraces of the Cu(111) surface under both dry and humid oxidation, in different directions. For humid oxidation, we also observed the formation of hydroxides and adsorbed water molecules after the Cu(111) surface was oxidized. We used near-ambient pressure X-ray photoelectron spectroscopy (NAP-XPS) to identify the oxidation states of the Cu and the related chemical species under reaction conditions. In addition, time-lapse NAP-XPS measurements revealed that both oxidations started as adsorbed oxygen atoms and proceeded to the Cu_2_O phase. The presence of water molecules induced the differences between humid and dry oxidation mechanisms; these differences were in the degree of reconstruction and surface coverage of the step edges on the oxidized surface. Finally, we interpreted the NAP-STM and NAP-XPS results to discuss how the presence of water vapor affected the oxidation mechanism and hindered additional subsurface oxidations.

## 2. Results and Discussion

### 2.1. In Situ Observations of Oxidations

**Figure 1:**
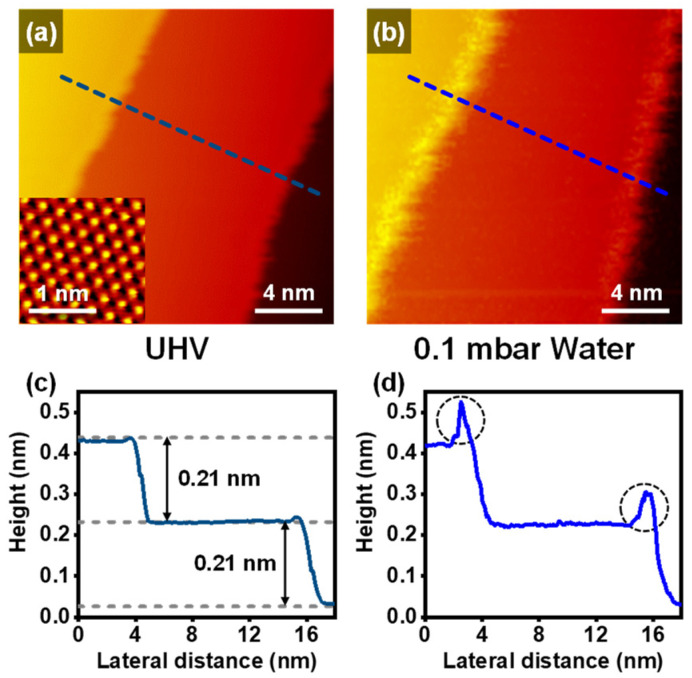
NAP-STM images of the Cu(111) (**a**) at UHV and RT (V_s_ = 0.71 V; I_t_ = 0.15 nA) with an atom-resolved STM image (Vs = 59 mV; It = 0.90 nA) shown in the inset, and (**b**) under 0.1 mbar of water (V_s_ = 0.78 V; I_t_ = 0.09 nA). (**c**,**d**) Representative height profiles for the dashed line in NAP-STM images of (**a**,**b**), respectively.

The prepared Cu(111) surface topography was observed by the NAP-STM at UHV and 298 K (RT) (Figure 1a). The inset in Figure 1a shows an atomic resolution image on the terrace with ~0.25 nm of the nearest-neighbor distance of the top sites. The hexagonal close-packed hollow sites (H_hcp_) and face-centered cubic hollow sites (H_fcc_) are also exhibited in the enlarged STM image (Figure S1 in the Supplementary Materials). The top site corresponds to the brightest spot in the STM image. The apparent height of H_hcp_ is slightly higher than that of H_fcc_. These STM images correspond to the surface structure of the fcc(111)-oriented copper surface [14]. In Figure 1c, the representative height profile indicates that the height of the monoatomic Cu(111) step is ~0.21 nm, which is well matched with other reference results [15,23].

Figure 1b shows the changes on the Cu(111) surface with 0.1 mbar of water vapor. The Cu(111) surface has no chemical interactions with water vapor even up to 1 mbar water conditions [5]. However, we observed protrusions, which appeared on the upper step edges. The representative line profile in Figure 1d exhibits that the apparent height of the protrusion is significantly higher than that of the clean step edges. The protrusions moved too fast to identify the stable structure of adsorbates, which means that the physisorption of water molecules occurred on the upper step edges. Even though the water molecules could induce the physisorption on the step edges, the Cu(111) surface kept its structure, including the step edges and terraces. Therefore, under near-ambient water gas conditions for the Cu(111), chemisorption did not occur, but weak physisorption happened.

**Figure 2:**
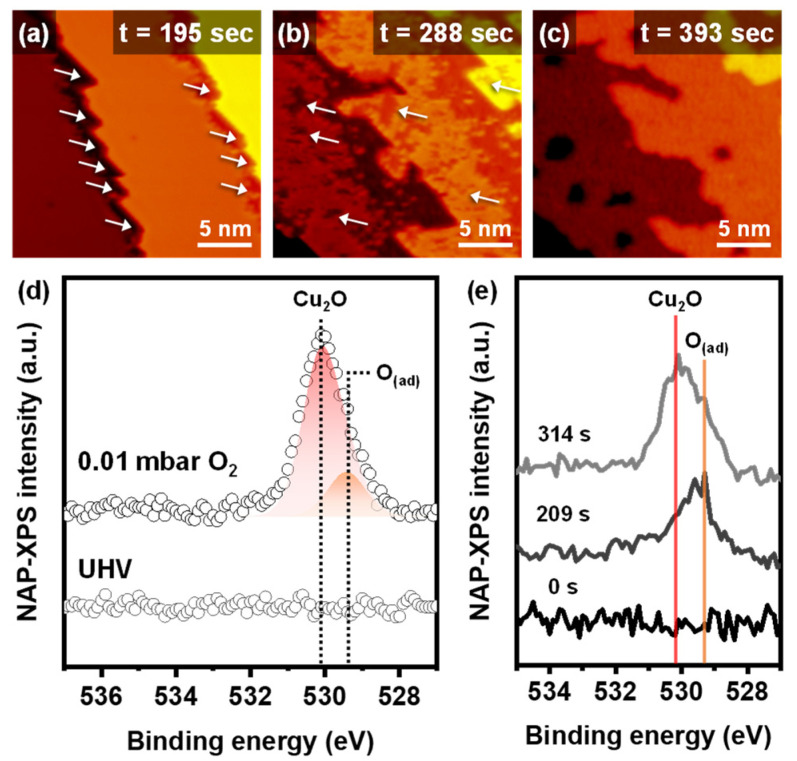
Time-lapse operando NAP-STM images of the dry oxidation process on Cu(111) surface at (**a**) 195 s, (**b**) 288 s (V_s_ = 0.76 V; I_t_ = 0.14 nA), and (**c**) 393 s (V_s_ = 0.71 V; I_t_ = 0.13 nA) under 0.01 mbar of O_2_ gas. (**d**) NAP-XPS operando core-level spectra for O 1s of clean Cu(111) at UHV and oxidized Cu(111) under 0.01 mbar of O_2_ gas. (**e**) Time-lapse NAP-XPS measurements of Cu_2_O and O_(ad)_ peaks evolution under 0.01 mbar of O_2_ gas.

Time-lapse changes of the Cu(111) surface under dry oxidation conditions are displayed in the operando NAP-STM images (Figure 2a–c and Figure S2). Dry oxidation started at the step edges under 0.01 mbar O_2_ gas conditions. In Figure 2a, the lower step edge sites, marked with white arrows, had a lower apparent height than the metallic Cu(111) terrace. The initial oxidation at the step edges is triangular, which was also observed in previous reports [14,15]. The apparent height of the copper oxide layer on the Cu(111) was lower than the metallic Cu(111) surface (Figure S2g–i), because of the combined contributions by the electronic and topographic effects [23,24]. Dry oxidation proceeded from the lower step edges to the terrace, and independent oxidation occurred on the terrace, as illustrated in Figure 2b. After enough exposure to the dry O_2_ gas environment, the surface was fully oxidized, and there were no further morphological changes under the same pressure. Figure 2c shows the fully oxidized Cu(111) surface with reconstructed step edges and terrace morphology. The fully oxidized Cu(111) surface had rough and collapsed step edges, while the metallic Cu(111) surface had straight step edges. Furthermore, holes formed on the oxidized Cu(111) terraces. The observations of dry oxidation show that the initial oxidation occurred at the lower step edges and the following oxidation induced the reconstruction of the surface morphology.

The operando NAP-XPS measurements were performed to identify the chemical species information during the dry oxidation of the Cu(111). Figure 2d demonstrates the XP spectra of O 1s at UHV and 0.01 mbar of O_2_ gas conditions. Analysis of the O 1s spectrum showed two deconvoluted peaks, cuprous oxides (Cu_2_O) and adsorbed oxygen atoms (O_(ad)_), at 530.2 eV and 529.4 eV, respectively [12]. The XP spectra of Cu 2p show the presence of only metallic copper and Cu_2_O (Cu^+^), not CuO (Cu^2+^), under every dry oxidation condition (Figure S4a). The peak area of Cu_2_O in the O 1s spectrum was dramatically larger than that of O_(ad)_, which indicates that the fully oxidized Cu(111) surface in the STM image consisted mainly of Cu_2_O phase with a few unstable adsorbed oxygen atoms. The time-lapse NAP-XPS shows the reaction steps for the dry oxidation (Figure 2e). At an initial stage of the dry oxidation, the O_(ad)_ appeared first. After that, only the intensity of the Cu_2_O peak increased until the Cu(111) surface was saturated. The time-lapsed XPS results match the NAP-STM images. The initial oxidation step was promoted by the O_(ad)_ produced by the dissociation of the O_2_ molecules on the lower step edges. Then, the O atoms reacted with Cu atoms to oxidize the Cu(111) terrace in the Cu_2_O phase. Therefore, the surface defects, including the step edge, are active sites for dissociating O_2_ molecules and oxidizing the Cu(111) surface [14,23].

**Figure 3:**
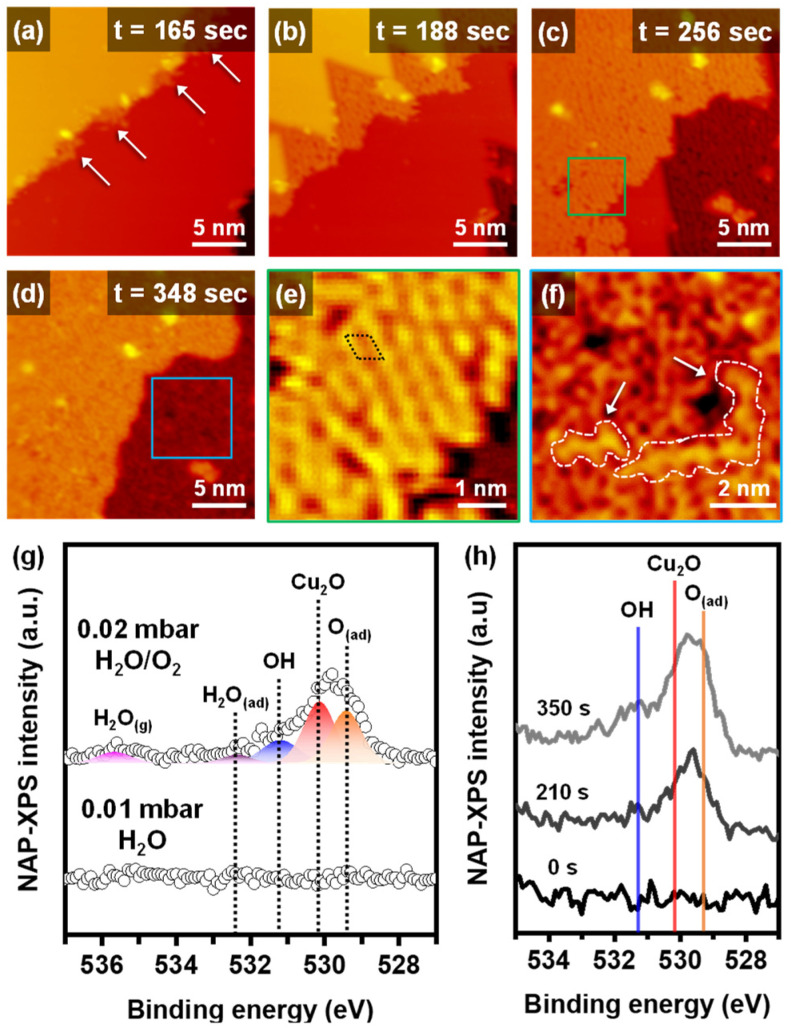
Time-lapse operando NAP-STM images of the humid oxidation process on Cu(111) surface at (**a**) 165 s, (**b**) 188 s (V_s_ = 0.88 V; I_t_ = 0.12 nA), (**c**) 256 s (V_s_ = 0.88 V; I_t_ = 0.13 nA), and (**d**) 348 s (V_s_ = 0.88 V; I_t_ = 0.14 nA) under 0.02 mbar of 1:1 ratio H_2_O/O_2_ gas mixture. Enlarged NAP-STM images of (**e**) a partially oxidized surface and (**f**) a fully oxidized surface. (**g**) NAP-XPS operando core-level spectra for O 1s of Cu(111) under 0.01 mbar of water and 0.02 mbar of H_2_O/O_2_ gas mixture. (**h**) Time-lapse NAP-XPS measurements of OH, Cu_2_O and O_(ad)_ peaks evolution under 0.02 mbar of H_2_O/O_2_ gas mixture.

Figure 3a–f and Figure S3 show the NAP-STM images under humid oxidation conditions of 0.02 mbar with an H_2_O/O_2_ 1:1 ratio gas mixture at RT. Similar to dry oxidation, the step edges were the starting point for humid oxidation, and then the oxidation proceeded to the metallic Cu terrace (Figure 3a–d). However, the formation of Cu_2_O started on the higher step edge, not the lower step edge, as seen in Figure 3a and Figure S3g–i. The existence of water molecules affected the initial oxidation site and reaction mechanisms. An enlarged STM image of a partially oxidized surface in Figure 3e shows a significantly different morphology from a fully oxidized surface, shown in an enlarged STM image in Figure 3f. The partially oxidized surface had an ordered hexagonal structure consistent with about 0.6 nm of an experimental lattice constant of Cu_2_O [15,17]. The hexagonal structure was also observed on the dry oxidized Cu(111) surface (Figure S8). When the Cu(111) surface was fully oxidized, the ordered structure almost disappeared, and a phase transition to disordered layers occurred (Figure 3f). The apparent height of the disordered array was larger than that of the ordered structure. The morphology of the disordered arrays changed continuously in each STM image, which was not observed on the dry oxidized surface. Zigzag H_2_O–OH chains were observed on an oxygen-precovered copper surface at low temperature (LT) by STM measurements [25,26]. The ordered zigzag water chain of LT and UHV condition can have a distorted structure, due to our experimental conditions of RT and NAP, as indicated by white arrows in Figure 3f.

We used the operando NAP-XPS measurements for the Cu(111) under humid oxidation conditions. The changes of chemical species on the O 1s XP spectra under 0.02 mbar of H_2_O/O_2_ (1:1 ratio) environment are displayed in Figure 3g. Under 0.01 mbar of water vapor conditions, no oxygen-related peaks were observed. Additionally, there were no significant changes on the other core-level spectra of C 1s (Figure S7b). When creating the H_2_O/O_2_ mixed gas conditions, the Cu(111) started to interact with the gas molecules. The O 1s spectrum can be distinguished as four different chemical species. The Cu_2_O and O_(ad)_ peaks were confirmed at the same position with dry oxidation at 530.2 eV and 529.4 eV, respectively. The additional deconvoluted peaks of the humid oxidation were hydroxides (OH) with a binding energy at 531.2 eV and adsorbed water molecules (H_2_O_(ad)_) with a binding energy at 532.4 eV [5,27]. There was also no Cu^2+^ peak observed in the Cu 2p spectra of the humid oxidation (Figure S4b). The humid oxidation process was observed by the time-lapse NAP-XPS in Figure 3h. Humid oxidation also started in the O_(ad)_ phase, and the surface was covered by the Cu_2_O phase. When the surface was saturated by the copper oxide species, the OH and H_2_O_(ad)_ peaks appeared on the spectrum. By clarifying the chemical species, we can explain the reaction steps of the humid oxidation. Humid oxidation started with the O_(ad)_ phase at the higher step edge and proceeded to the terrace without independent oxidation. Before the Cu_2_O fully covered the surface, no OH and H_2_O_(ad)_ peaks were observed in NAP-XP spectra, which matched NAP-STM images well, demonstrating that the ordered hexagonal structures of Cu_2_O disappeared, and the disordered array appeared on the top surface. Water molecules preferred to be dissociated when oxygen was preadsorbed on copper surfaces [28,29,30]. In addition, strong hydrogen bonds induced H_2_O–OH complexes to be stable as a final state of autocatalytic water dissociation on Cu(110) [31].

### 2.2. Surface Oxidation Mechanisms of Dry and Humid Oxidation

**Figure 4:**
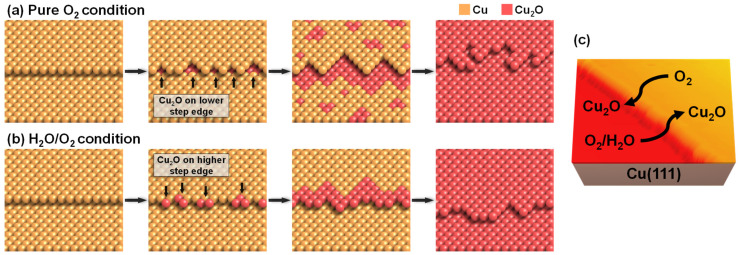
Schematic images of reaction mechanisms for (**a**) the dry oxidation and (**b**) the humid oxidation. (**c**) 3-dimensional representative NAP-STM image showing the initial oxidation sites of Cu(111) surface.

The experimental results confirmed that the presence of water molecules affected the reaction mechanisms and the final states of the oxidized Cu(111) surface. Figure 4 represents the schematic images for the dry and humid oxidation processes. For dry oxidation, only oxygen gas molecules interacted with the surface defects of Cu(111). Specifically, the lower step edge was the reactive site to dissociate oxygen molecules directly to oxygen atoms. It is a well-known phenomenon that electron charge density is different at the higher and lower side of the step edge, due to the Smoluchowski effect [32]. Additionally, the results of Xu and Mavrikakis demonstrated that the molecular O_2_ precursor preferred the electron-rich lower step edge by the density functional theory (DFT) calculations [33]. Therefore, it is not surprising that dry oxidation initiated at the lower step edge of the Cu(111) surface. The next dry oxidation steps were the sequential oxidation of the lower step oxidation, progressing to the terrace and the independent terrace oxidation (Figure 4a). The dissociative O atoms diffused from the step edge to the terrace to form Cu_2_O superstructures, which indicates that the sequential dry oxidation occurred at the boundaries between Cu metal and Cu_2_O phase [24]. High-resolution in situ transmission electron microscopy images of a Cu(110) surface show the oscillatory evolution of the Cu_2_O layer, explaining the Cu_2_O propagation from an upper Cu terrace to a downward edge and flattening a stepped surface [34]. Similarly, the independent oxidations were started by the diffusion of oxygen atoms to the terrace, which were not incorporated in the sequential step oxidation. Previous DFT calculation results showed that the dissociative O atoms preferred to move away from each other and be stabilized at distant terrace sites of the stepped Cu(211) surface [33], which made the formation of independent terrace oxides possible. After the Cu_2_O regions formed on the Cu(111) surface, the direct impingement and surface diffusion of the O atoms on the metal-oxide interfacial surface existed to grow the Cu_2_O layer [13,35].

Under humid oxidation conditions, the initial oxidation was observed on the higher step edge. The coexistence of water molecules with O_2_ molecules induced the humid oxidation process differently from dry oxidation (Figure 4b). While water molecules adsorbed at the upper step edge (Figure 1b), O_2_ molecules preferred to occupy the lower step edge. The adsorption of water molecules on the upper step edge of metal surfaces was reported in other literature [36,37]. Because of the Smoluchowski effect, the higher step edge has reduced electron charge density, having the higher density of unoccupied states, which attracts water molecules to be adsorbed [38]. Although the 1D water chains and 2D networks were investigated under low temperature conditions, below ~200 K [39,40,41], our observation results at RT do not show any stable water structures. When water and O_2_ coexisted on the Cu(111) surface, copper oxides formed using oxygen from water molecules at 65 °C for 30 days [42]. Thus, we assume that the reaction mechanism of the humid oxidation in our experiments can include the dissociation of water molecules. As seen in Figure 3h, humid oxidation also initiated at the O_(ad)_ phase. This indicates that the generation of oxygen atoms was the first step for humid oxidation. It was more stable for water molecules to attract oxygen atoms than to separate in an infinite range [43]. Thus, the dissociative O atoms produced Cu_2_O by reacting with water molecules, not directly oxidizing the metallic copper on the upper step edges of the Cu(111). No independent oxidations on the terrace were observed, also verifying that the water interaction with oxygen atoms is crucial for the humid oxidation mechanism. However, in our investigation, we cannot confirm that all of the Cu_2_O originated from oxygen atoms of water molecules. Our humid oxidation reaction conditions at RT for a few hours were different from the previous study [42]. Although we cannot confirm the total origin of oxygen, water molecules certainly contribute to the humid oxidation mechanisms by interacting with dissociative oxygen atoms.

### 2.3. Different Thickness of the Cu_2_O by the Subsurface Oxidations

**Figure 5:**
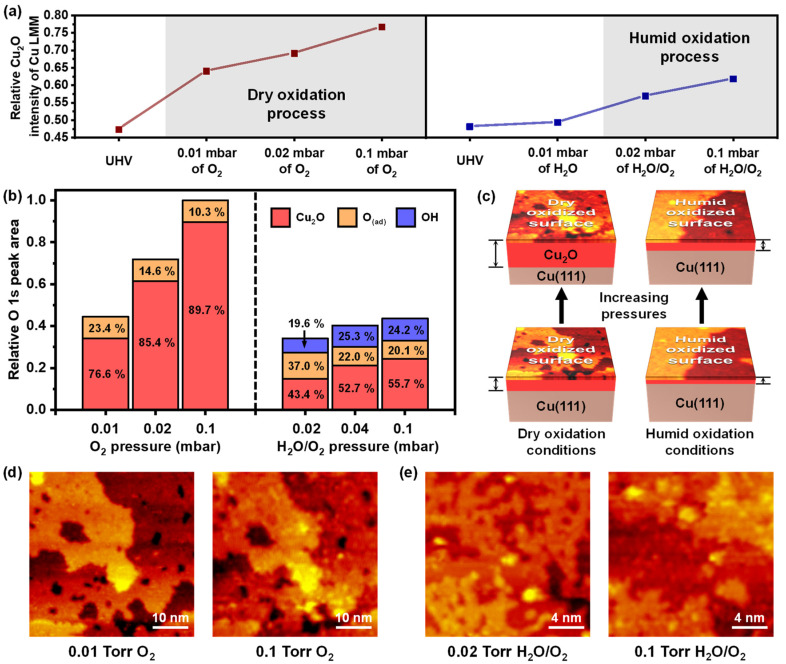
(**a**) Relative peak intensity histogram of Cu_2_O/Cu ratio from Cu LLM spectra under different oxidation reaction conditions. (**b**) Relative peak area histogram of O 1s NAP-XPS core-level measurements under dry and humid oxidation process. (**c**) Schematic representations of Cu(111) oxidation process for the surface fully covered by dry oxidized surface under increased O_2_ gas pressure and covered by humid oxidized surface under increased H_2_O/O_2_ gas mixture pressure. (**d**,**e**) NAP-STM images under different oxidation conditions used for surface STM images in (**c**).

Figure 5a,b show the histograms of numerical analysis for both oxidations with increasing pressures. The photoemission spectra of the Cu LMM were plotted as the trends of the relative peak intensities for the Cu_2_O/Cu ratio (Figure 5a). The representative NAP-XAES peaks for Cu_2_O and Cu 916.6 eV and 918.6 eV, respectively, are seen in Figure S6 [1,44,45]. We excluded the possibility of the CuO, because the Cu^2+^ peaks were not taken in the Cu 2p XP spectra (Figure S4). The relative XAES Cu_2_O intensities of dry oxidation were significantly greater, even when the partial pressure of O_2_ gas of the humid oxidation was higher. The well-matched results of O 1s XP spectra were proposed in Figure 5b, demonstrating that the total O 1s peak areas were always larger for dry oxidation than for humid oxidation. The comparison with O 1s peak areas was conducted on the raw XP spectra without any normalization of peak intensities (the trend was consistent even with the normalization). Interestingly, the Cu_2_O areas increased sequentially with the higher total pressures under both oxidation conditions, even though the Cu_2_O peak areas of the dry oxidation were significantly larger than those of the humid oxidation under every pressure condition. The topmost Cu_2_O layer already covered the entire surface of the Cu(111) in 0.01 mbar of pure O_2_ conditions and 0.02 mbar of H_2_O/O_2_ mixed conditions (Figures S2 and S3).

The further formation of Cu_2_O required another metallic Cu, which was not previously present on the surface. We propose two pathways to generate the additional oxidation: (i) the outward diffusion of the subsurface copper atoms and (ii) the inward diffusion of the dissociative O atoms. The copper atoms’ diffusion to the surface was regarded as a potential way to activate the growth of the Cu_2_O layer. The Cu_2_O is known as a p-type semiconductor, inducing a Cu cation to move into an oxide–air interface of the oxidized Cu(111) surface [46,47]. Although Cu diffusion is more difficult at RT than high temperature, we directly observed Cu_2_O formation, induced by the outward diffusion of the sublayer Cu with increasing the pressure of pure O_2_ gas and H_2_O/O_2_ gas. The black arrows of representative height profiles of Figures S9 and S10 indicate the newly formed Cu_2_O structures on the identical regions obtained by the NAP-STM. The higher pressure motivates Cu migration from the sublayer to the surface, including a horizontal diffusion [48]. However, since the additional formation of Cu_2_O occurred locally, it does not fully explain the dramatic Cu_2_O increment in XP spectra. Thus, we consider the inward diffusion of the O atoms as another factor of the Cu_2_O peak area increments. The penetration of the O atoms toward the subsurface was discovered experimentally by a thermal oxidation above 500 K of the Cu(111) [49] and collision-induced adsorption with a hyperthermal O_2_ molecular beam at RT [50]. The inward O atom diffusion was possible under our experimental conditions at RT with high pressure above 0.01 mbar of O_2_ gas, because of a free energy difference induced by a pressure gap [51]. By enhancing chemical potential, the number of collisions for O_2_ molecules with the surface was increased to supply additional O atoms [52]. As oxidized, the Cu(111) surface reconstructed to migrate the O atoms into the subsurface. After the Cu(111) surface was fully covered by the Cu_2_O, an interfacial diffusion of the oxygen proceeded to induce the epitaxial growth of the copper oxide [13,53]. Therefore, the mixed effect of the two oxidation pathways, (i) and (ii), promoted the additional oxidation, and the Cu_2_O thickness became larger with the increased pressure (Figure 5c). The surface STM images in Figure 5c are shown in Figure 5d,e, and their parameters are presented in Figures S9 and S10.

The degree of oxidation was larger for dry oxidation than humid oxidation, as mentioned above (Figure 5a,b). The difference in the oxidation depth was due to the difference in degree of the additional oxidation. The additional oxidation by the mixed pathways with a continuous O_2_ exposure causes the rough Cu_2_O surface to coalesce and form a smooth surface [13]. Thus, we assume that further oxidation proceeds actively until the Cu_2_O surface becomes flat without surface defects; an ultraflat Cu(111) surface without atomic steps is oxidation resistant after more than a year of air exposure [54]. Therefore, the step edges and surface defects are essential to promote the oxidation of Cu(111). Figure S11 demonstrates that the surface coverage of the step edge on the Cu_2_O surface was more significant for dry oxidation, because the Cu(111) surface had different oxidation mechanisms, depending on the presence or absence of water molecules. Therefore, the additional oxidation from crossing diffusion of Cu and O atoms occurred more actively for the dry oxidized surface with larger coverage of the defects than the humid oxidized surface. Furthermore, as the depth of Cu_2_O increased, the migration of Cu to the surface became a dominant factor for additional oxidation [46]. However, the OH layers on the humid oxidation could hinder the oxidized Cu(111) from the formation of the subsurface oxides. The water molecules can interact with the Cu ion that migrates from the subsurface to form OH and water complexes. In addition, a metastable Cu(OH)_2_ phase transforms to a stable CuO phase under humid conditions at RT, which uses the hydroxides from the water molecules [42,46], even though we observed no Cu(OH)_2_ peak in the XPS spectra, because of the shorter reaction time than that of the references. In other words, the outward diffused Cu reacted competitively with the O_2_ and water molecules, which reduced the possibility for additional oxidation. We did not observe the saturation of both oxidations because of the mild oxidation conditions (NAP and RT) and the short exposure times, which exceeded the probing depth of the XPS and XAES.

We confirmed how the hydrophilicity of Cu(111) developed under humid O_2_ conditions by operando observation techniques. The evolution of the Cu_2_O layer was directly observed under dry and humid conditions to explain the different reaction mechanisms affecting the degree of oxidation, providing a fundamental understanding of many catalytic reactions utilizing the copper-based materials, oxygen, and water gas molecules. The oxidation process from metallic Cu(111) to Cu_2_O was important for analyzing the CO oxidation mechanism [12]. For the water gas shift reaction, the Cu–Cu_2_O suboxide interface was the active site for water dissociation to react with CO molecules [7]. Finally, water molecules and the total pressure conditions could limit the copper material from rapid oxidation. By controlling the humidity and pressure of oxidation environments, the degree of oxidation can be adjusted, which influences the performance of catalysis. For example, the thickness of Cu_2_O layer is crucial for the electroreduction of CO_2_ via the changing electroactive surface areas [55]. Therefore, our results in this work would enhance the possibility of investigating catalytic reactions by controlling the Cu_2_O depth under even ambient pressure conditions.

## 3. Materials and Methods

### 3.1. Preparation of Cu(111) Single Crystal Surface

A commercially available Cu(111) single crystal was purchased from Mateck GmbH (Jülich, Germany). The Cu(111) single crystal had a high cut accuracy < 0.1° and one-side polished surface. A well-ordered Cu(111) surface was prepared by sample cleaning cycles by Ar^+^ ion-bombardment sputtering (P_Ar_ = 1 × 10^−5^ mbar at 1.5 keV) and vacuum annealing at 900 K for 5 min. The sample cleaning procedure was repeated until obtaining a contaminant-free and well-ordered Cu(111) surface, which was confirmed using STM and XPS measurements.

### 3.2. Operando Observations Using NAP-STM

A reaction cell-integrated STM scanner (Aarhus STM 150 NAP, SPECS GmbH, Berlin, Germany) in a UHV analysis chamber (base pressure: 1 × 10^−10^ mbar) of a NAP-STM system was used to acquire operando NAP-STM images [56,57]. The inside volume (15 mL) of the reaction cell was physically separated from the UHV chamber by o-rings and locking screws. A multi-gas delivery manifold system introduced high-purity O_2_ (99.999%) and ultrapure water gas molecules to the reaction cell. Ultrapure water was degassed by repeated freeze-pump-thaw cycles to remove impurities. The pressures of near-ambient conditions were measured by a full-range gauge (Pfeiffer Vacuum GmbH, Aßlar, Germany) connected to the reaction cell. All topographic STM images were taken by a chemically etched tungsten tip at RT (298 K). A constant current mode was used to record the STM images, and tunneling parameters are denoted as V_s_ and I_t_ for sample bias voltage and tunneling current, respectively.

### 3.3. Operando Observations and Analysis Using NAP-XPS

The NAP-XPS measurements for the Cu(111) oxidation reactions were performed at the Advanced in situ Surface Analysis System (AiSAS) of the Korea Basis Science Institute (KBSI) in the Republic of Korea [58,59]. X-ray photoemission spectra were acquired into an operando high-pressure reaction cell integrated in the NAP-XPS system. One X-ray source was Al Kα with photon energy 1486.74 eV. A PHOIBOS 150 NAP hemispherical electron analyzer (SEPCS) was used to measure the X-ray photoemission spectra. All gas lines were baked out at 400 K with a diaphragm pump and a turbomolecular pump (Pfeiffer Vacuum GmbH, Aßlar, Germany) for 12 h before O_2_ gas and water vapor were introduced to the NAP-XPS system. The freeze–pump–thaw cycles were repeated until no contaminant carbon species were detected by the NAP-XPS measurements for water gas. The collected XP spectra were recorded with an energy step of 0.1 eV and a pass energy of 40 eV at 298 K. The Fermi edge of the Cu(111) was used to calibrate the binding energy of the spectra. The XP spectra were subtracted by a Shirley-type background and O 1s XP spectra were deconvoluted with a widely accepted mixed function of a Gaussian (70%) and Lorentzian (30%). Each the XP spectra analysis was conducted using the CasaXPS package. The X-ray induced Auger electron spectroscopy (XAES) results were also acquired in the same condition with the NAP-XPS.

## 4. Conclusions

The Cu(111) oxidation processes have been observed by operando NAP-STM and NAP-XPS at RT. The pure water vapor affected no chemical changes in the Cu(111) topmost structure, except the physisorption on the step edges. Dry oxidation under 0.01 mbar of O_2_ gas initiated at the lower step edges with independent oxidation on the terrace. Under 0.02 mbar of O_2_/H_2_O gas mixture, humid oxidation proceeded from the higher step edges to the terrace without independent oxidation, explaining that water molecules certainly participated in the oxidation mechanisms. For both the oxidation processes, the oxidation started as the O_(ad)_ followed by the Cu_2_O species. After the Cu(111) surface was fully oxidized, the OH and H_2_O related species were formed only for the humid oxidation. The differences of the oxidation mechanisms induced by the presence of water molecules caused the dry oxidized sample to have a rougher surface. When the Cu_2_O phase entirely covered the surface, the additional oxidation occurred, due to the outward diffusion of the copper and the inward diffusion of the oxygen. The additional oxidations reconstructed the oxidized surface and the metallic subsurface, producing the larger thickness of the Cu_2_O layer. Finally, the dry oxidized Cu(111) had a thicker Cu_2_O layer than the humid oxidized Cu(111) under similar exposure pressures and times, because of the roughness and the different chemical species of the surface. Our investigations confirm that the presence of water molecules changes the initial surface oxidation mechanism and lowers the degree of the oxidation progress.

## Data Availability

Not applicable.

## Supplementary Materials

The following supporting information can be downloaded at: https://www.mdpi.com/article/10.3390/ijms24010810/s1.
