# Supplementary material for: Effect of Water Vapor on Oxidation Processes of the Cu(111) Surface and Sublayer
Source: Int J Mol Sci. 2023 Jan 3;24(1):810. doi: 10.3390/ijms24010810 (PMC9821670; doi:10.3390/ijms24010810)
Supplement: Supplementary file 1 [file ijms-24-00810-s001.zip › ijms-2108785-supplementary.pdf]

Supporting Information

Effect of Water Vapor on Oxidation Processes of the Cu(111) Surface and Sub-Layer

Young Jae Kim[1,2], Daeho Kim[1,2], Yongman Kim[1,2], Yongchan Jeong[1], Beomgyun Jeong[3] and Jeong Young Park[1,2,*]

[1] Department of Chemistry, Korea Advanced Institute of Science and Technology (KAIST), Daejeon 34141, Republic of Korea

[2] Center for Nanomaterials and Chemical Reactions, Institute of Basic Science (IBS), Daejeon 34141, Republic of Korea

[3] Research Center for Materials Analysis, Korea Basic Science Institute (KBSI), Daejeon 34133, Republic of Korea

* Correspondence: jeongypark@kaist.ac.kr

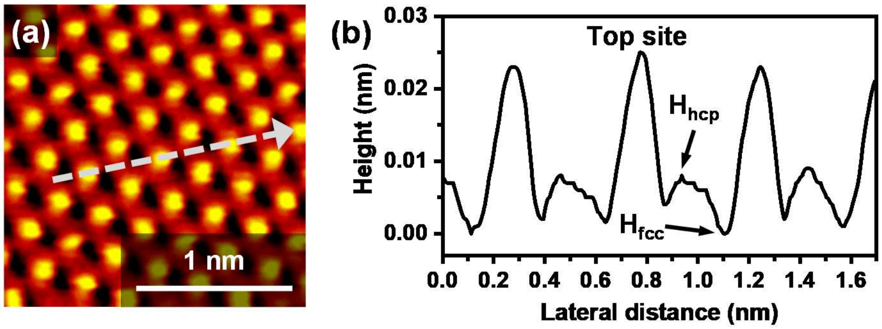

Figure S1. (a) Atomic−resolved NAP-STM image of the Cu(111) at UHV. (b) Representative height profile of the Cu(111) surface.

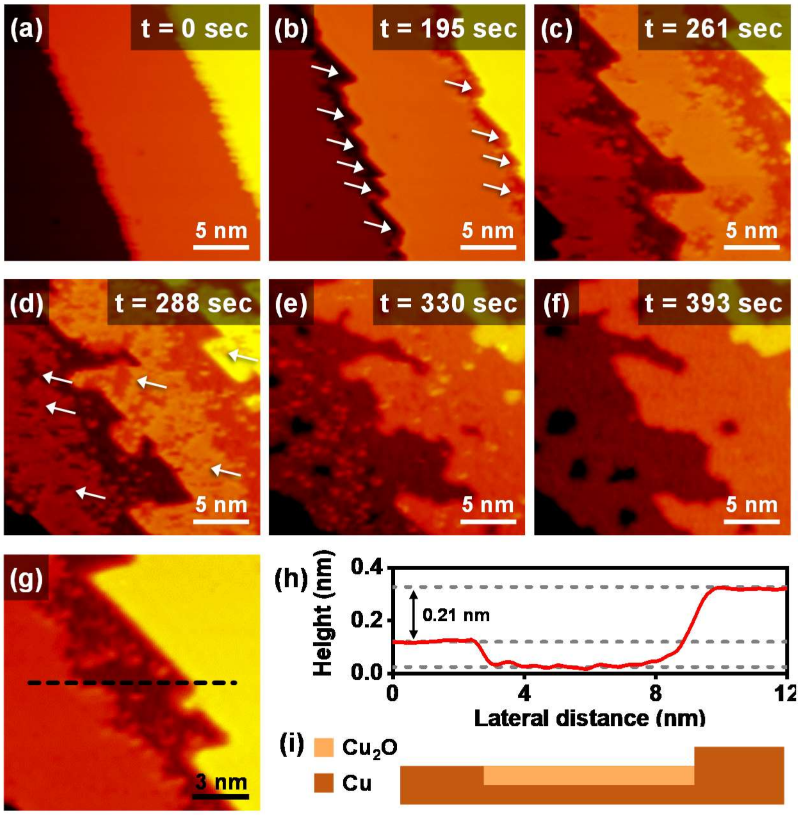

Figure S2. Time-lapse operando NAP-STM images of Cu(111) surface under 0.01 mbar of O_2 gas at (a) 0 sec, (b) 195 sec, (c) 261 sec, (d) 288 sec, (e) 330 sec, and (f) 393 sec ($V_s = 0.71 - 0.76$ V; $I_t = 0.13 - 0.15$ nA). (g) Enlarged NAP-STM image during the dry oxidation, (h) representative height profile of (g), and (i) schematic image of surface morphology corresponding with the height profile.

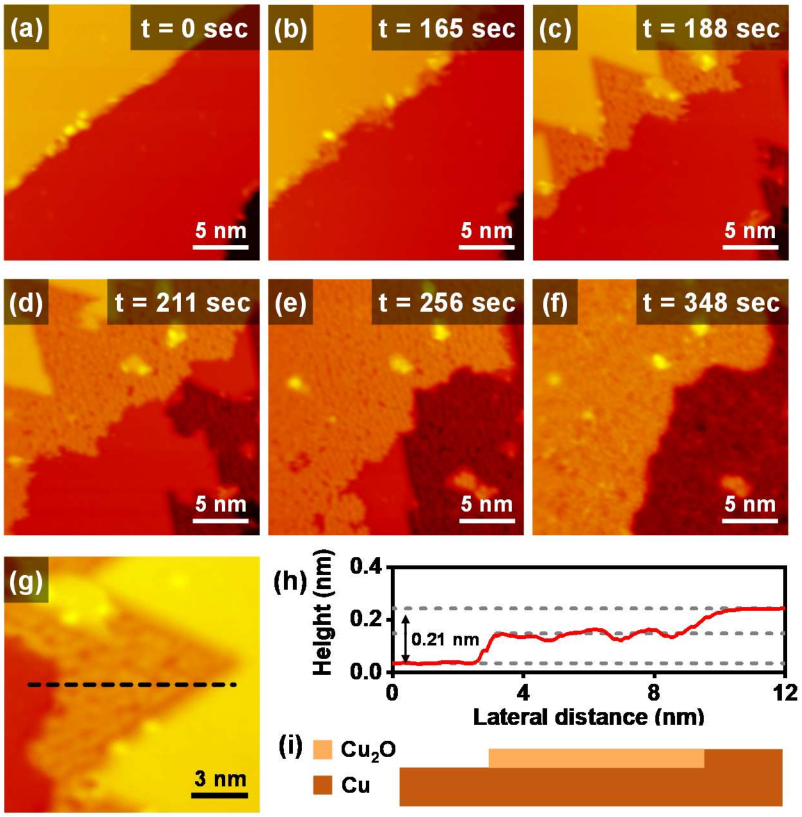

Figure S3. Time-lapse operando NAP-STM images of Cu(111) surface under 0.02 mbar of H_2O/O_2 gas mixture at (a) 0 sec, (b) 165 sec, (c) 188 sec, (d) 211 sec, (e) 256 sec, and (f) 348 sec ($V_s = 0.81 - 0.88$ V; $I_t = 0.12 - 0.14$ nA). (g) Enlarged NAP-STM image during the humid oxidation, (h) representative height profile of (g), and (i) schematic image of surface morphology corresponding with the height profile.

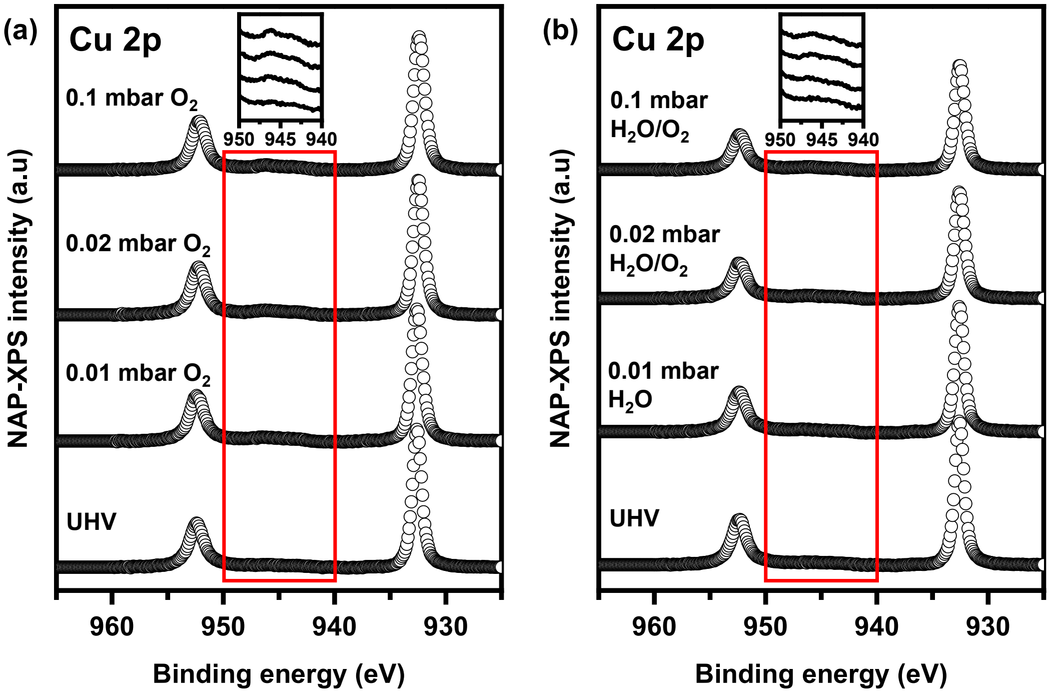

Figure S4. NAP-XPS operando core−level spectra for Cu 2p of Cu(111) at RT (a) under O_2 gas conditions and (b) 1:1 ratio H_2O/O_2 gas mixture conditions.

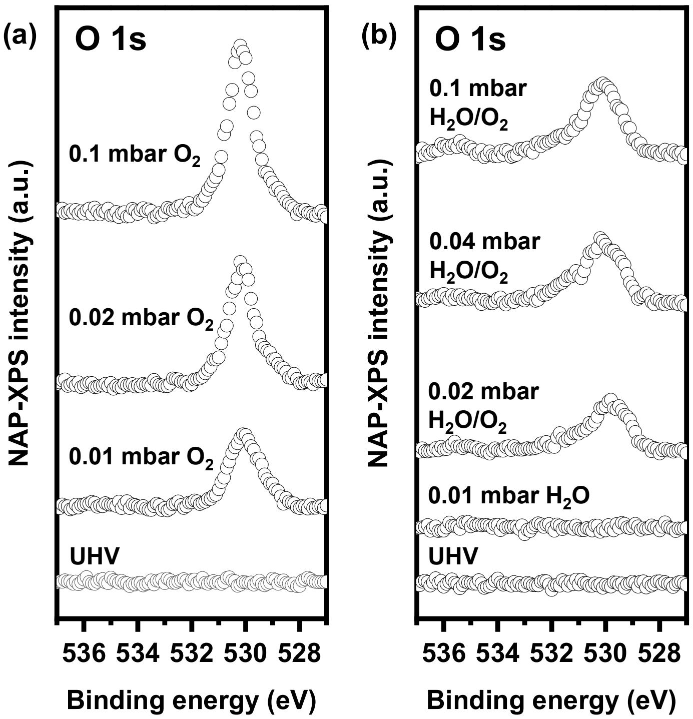

Figure S5. NAP-XPS operando core−level spectra for O 1s of Cu(111) at RT (a) under O_2 gas conditions and (b) 1:1 ratio H_2O/O_2 gas mixture conditions.

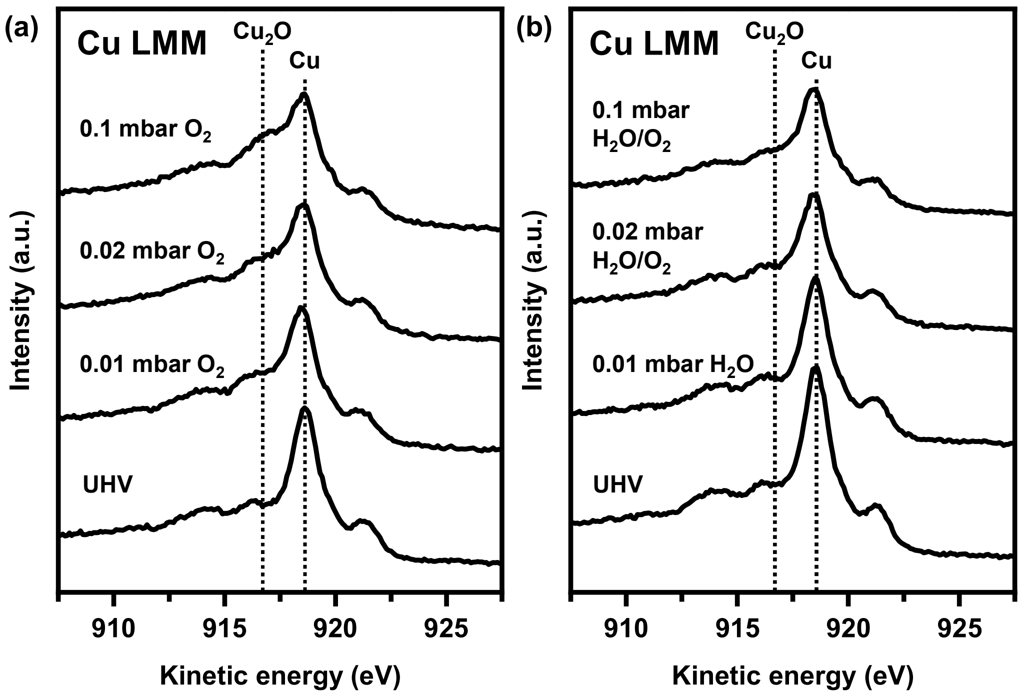

Figure S6. Operando XAES for Cu LMM of Cu(111) at RT (a) under O_2 gas conditions and (b) 1:1 ratio H_2O/O_2 gas mixture conditions.

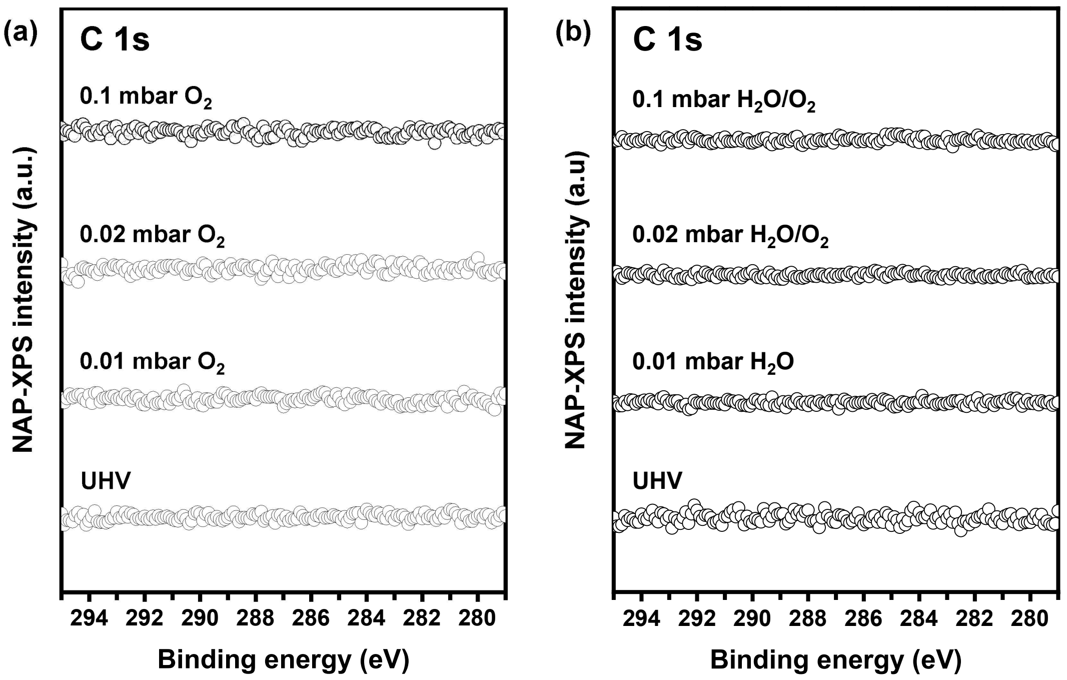

Figure S7. NAP-XPS operando core−level spectra for C 1s of Cu(111) at RT (a) under O_2 gas conditions and (b) 1:1 ratio H_2O/O_2 gas mixture conditions.

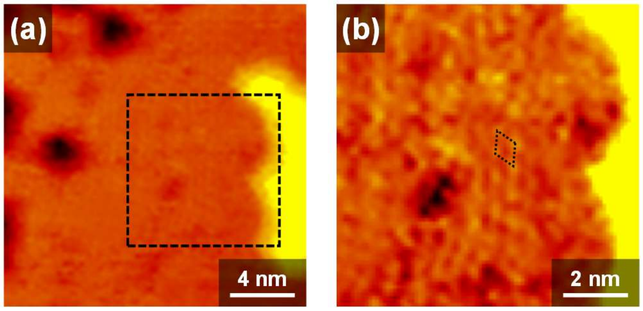

Figure S8. (a) A NAP-STM image of O/Cu(111) at RT under 0.03 mbar of O_2 gas and (b) an enlarged NAP-STM image of the dashed square region (V_s = 0.59 V; I_t = 0.12 nA).

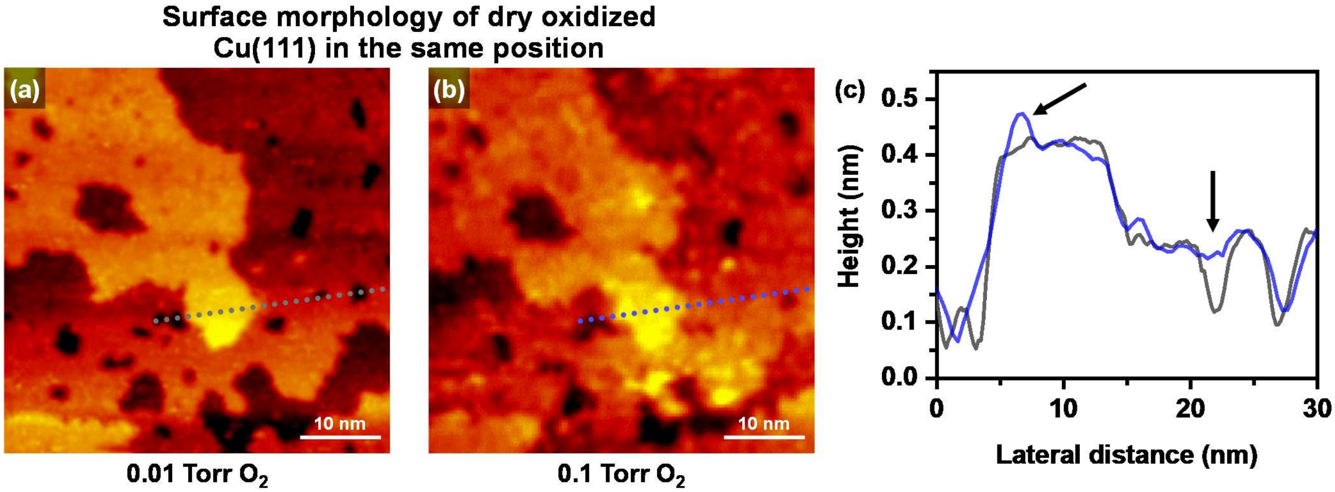

Figure S9. NAP-STM images of dry oxidized Cu(111) at same position and RT under (a) 0.01 mbar of O_2 gas (V_s = 0.65 V; I_t = 0.15 nA) and (b) 0.1 mbar of O_2 gas (V_s = 0.63 V; I_t = 0.16 nA). (c) Representative height profile for the dashed lines in (a) and (b).

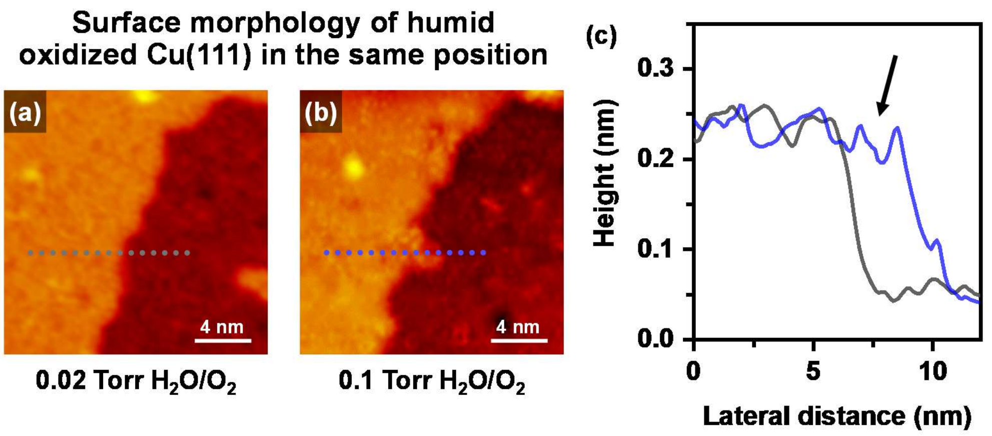

Figure S10. NAP-STM images of humid oxidized Cu(111) at same position and RT under (a) 0.02 mbar of H_2O/O_2 gas mixture (V_s = 0.88 V; I_t = 0.14 nA) and (b) 0.1 mbar of H_2O/O_2 gas (V_s = 0.86 V; I_t = 0.14 nA). (c) Representative height profile for the dashed lines in (a) and (b).

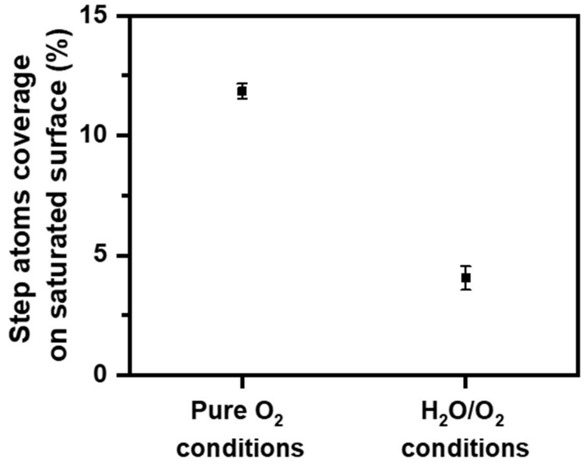

Figure S11. Coverages of step atoms on saturated Cu(111) surface under different oxidation conditions, which are acquired by NAP-STM images. In pure O_2 conditions, the pressure ranges from 0.01 mbar to 0.1 mbar of O_2 gas are included to calculate the step atoms coverages. Similarly, in H_2O/O_2 conditions, the pressure ranges from 0.02 mbar to 0.2 mbar of H_2O/O_2 (1:1) gas mixture are included to calculate the step atom coverages.
